# IL-6 and TGF-β1 as biomarkers of schistosomiasis-associated pulmonary hypertension in a murine model

**DOI:** 10.1590/1414-431X2025e15221

**Published:** 2026-01-30

**Authors:** A.C. Roque, I.S. Stockmann, M.M.P. Acencio, C.S.R. Silva, M.C. do Espírito-Santo, P.L.S. Pinto, T. Mauad, M.C. Irigoyen, R.O. Crajoinas, R. Souza, W. Salibe-Filho

**Affiliations:** 1 Divisão de Pneumologia, Instituto do Coração (InCor), Hospital das Clínicas (HCFMUSP), Faculdade de Medicina, Universidade de São Paulo, São Paulo, SP, Brasil; 2 Instituto de Medicina Tropical, Faculdade de Medicina, Universidade de São Paulo, São Paulo, SP, Brasil; 3 Centro de Parasitologia e Micologia, Instituto Adolfo Lutz, São Paulo, SP, Brasil; 4 Departamento de Patologia, Faculdade de Medicina, Universidade de São Paulo, São Paulo, SP, Brasil; 5 Laboratório de Hipertensão Experimental, Instituto do Coração (InCor), Hospital das Clínicas, Faculdade de Medicina, Universidade de São Paulo, São Paulo, SP, Brasil; 6 Divisão de Cardiologia, Instituto do Coração (InCor), Hospital das Clínicas, Faculdade de Medicina, Universidade de São Paulo, São Paulo, SP, Brasil

**Keywords:** Pulmonary arterial hypertension, Schistosomiasis, Inflammatory markers, Experimental model

## Abstract

Schistosomiasis can lead to vascular damage resulting in pulmonary arterial hypertension (PAH). Although its pathophysiology remains unclear, cytokine imbalance is known to play a key role. This study aimed to evaluate serum mediators in association with hemodynamic, echocardiographic, and histological parameters in a murine model of *Schistosoma mansoni*-induced pulmonary hypertension (Sch-PH). Twenty male C57BL/6 mice were randomized into infected group and non-infected control group. Sch-PH was induced by intraperitoneal inoculation of *S. mansoni* eggs (240 eggs/g body weight), followed by intravenous administration (175 eggs/g). After 21 days, systolic pulmonary artery pressure was measured by right ventricular catheterization (RHC), and cardiac function was assessed by transthoracic echocardiography. Animals were then euthanized for collection of lungs and heart for histopathology, and blood samples were obtained for quantification of interleukin (IL)-6, IL-10, and tumor necrosis factor (TGF)-β1 by ELISA. The Sch-PH group had significantly lower tricuspid annular plane systolic excursion and pulmonary artery acceleration time/pulmonary ejection time ratio (P<0.05), and increased pulmonary artery peak flow, tricuspid and pulmonary regurgitation, IL-6, and TGF-β1 levels (P<0.05). IL-10 was undetectable. Lung tissue showed inflammatory infiltrates, alveolar and perivascular granulomas, and *S. mansoni* eggs. Pulmonary arteries exhibited intimal thickening, medial hypertrophy, and fibrosis. Cardiac tissue presented inflammatory foci, fibroblast proliferation, and thickening of connective septa. IL-6 and TGF-β1 were elevated in Sch-PH and correlated with echocardiographic and hemodynamic alterations. These findings suggest a role for these mediators in Sch-PH pathogenesis and highlight the potential for targeting inflammatory pathways in this condition.

## Introduction

Schistosomiasis, caused by the trematode *Schistosoma mansoni*, can induce vascular injury and pulmonary circulation remodeling, potentially leading to pulmonary arterial hypertension (PAH) (1,2). Despite its clinical significance, the pathophysiological mechanisms underlying this chronic complication remain incompletely understood (3). Initially, pulmonary egg deposition was thought to be the primary driver of vascular obstruction, but the extent of this physical obstruction alone is insufficient to explain the development of PAH (3). Recent evidence indicates that, in addition to egg presence, schistosomiasis-associated pulmonary hypertension (Sch-PH) involves an inflammatory response that has not yet been fully characterized (4). Understanding these mechanisms is crucial for the medical and scientific community, as it may guide early diagnosis, risk stratification, and the development of targeted therapeutic strategies, ultimately improving outcomes for patients in endemic regions and contributing to broader insights into pulmonary vascular disease.

Some authors suggest that the inflammatory response and inflammatory pathways play important roles in the pathogenesis of PAH (5). Large infiltrates of inflammatory cells around the blood vessels of pulmonary arteries have been observed in patients and animal models of PAH (6,7). The balance between inflammatory and anti-inflammatory cytokines plays a vital role in the pathogenesis of PAH (8). Savale et al. (9) suggest that IL-6 promotes the development and progression of pulmonary vascular remodeling and PAH through proproliferative antiapoptotic mechanisms.

In another study, Ito et al. (8) noted that the expression of transforming growth factor-β1 (TGF-β1) and interleukin (IL)-6 in lung tissue was reduced by IL-10, reporting possible preventive effects by influencing factors that play essential roles in the progression of PAH remodeling. A more recent study showed an IL-6 increase in pulmonary hypertension in heart transplant patients (10).

This study aimed to characterize serum IL-6, IL-10, and TGF-β1 levels and their relationship with hemodynamic and histopathological alterations in an experimental model of Sch-PH, providing mechanistic insights into disease pathogenesis and identifying potential biomarkers of progression.

## Material and Methods

### Animals

Twenty male C57Bl/6 mice (6-8 weeks old) were obtained from the University of São Paulo/School of Medicine Laboratory Animal Center. The University Ethics Committee (CEUA 1594/2021) approved animal care, methods, and experimental procedures.

### *Schistosoma mansoni* eggs

*Schistosoma mansoni* eggs were harvested from homogenized and purified livers of golden hamsters (*Mesocricetus auratus*) infected with cercariae, provided by the Schistosomiasis Laboratory of the Center for Parasitology and Mycology, Adolfo Lutz Institute (Brazil).

Immediately following isolation, eggs were carefully washed with sterile saline to remove tissue debris and minimize contamination. They were then maintained under controlled conditions at 4°C for a brief period prior to their use in sensitization and challenge, with storage time not exceeding 1 h to preserve maximum viability.

### Experimental design

**Figure 1:**
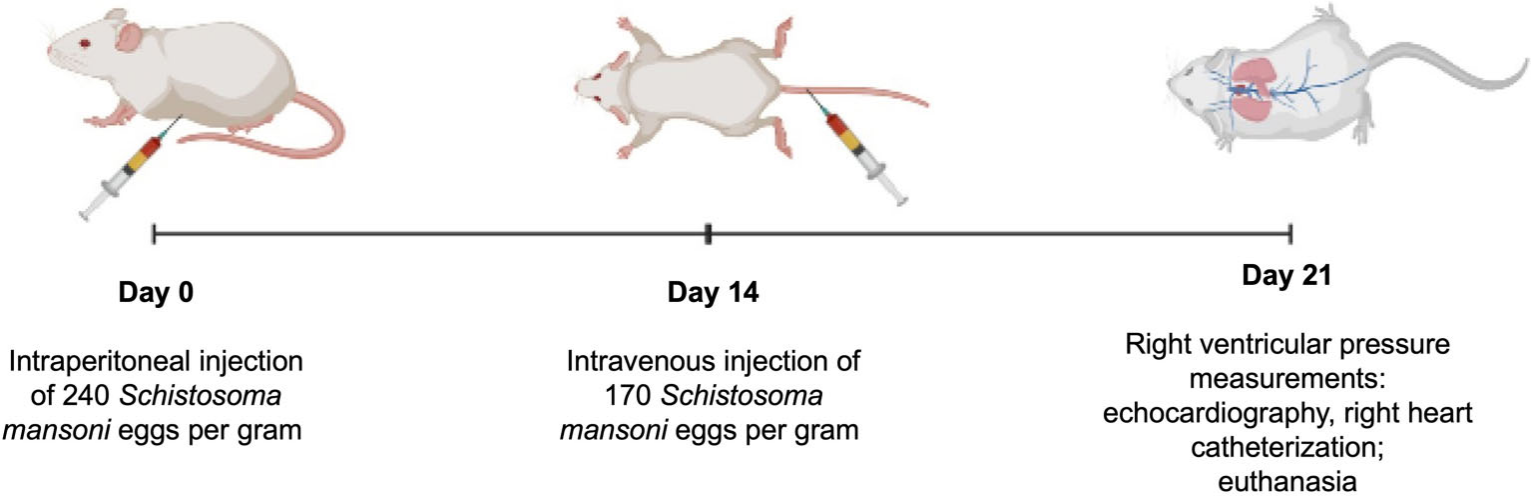
Diagrammatic representation of the experimental design.

The animals were randomly divided into two groups (10 animals each). The experimental mice were intraperitoneally (*ip*) sensitized with 240 *S. mansoni* eggs/g body weight and, two weeks later, intravenously (*iv*) challenged with 175 *S. mansoni* eggs/g body weight. Control mice were unexposed to *S. mansoni* eggs (Figure 1) (11).

After 21 days, the animals were evaluated for the development of vascular disease, using echocardiographic and invasive measurements through catheterization to assess right ventricular systolic pressure (RVSP).

The animals were anesthetized with 2% isoflurane delivered in 95% oxygen, and animals were maintained under anesthesia using the same concentration throughout all procedures. Vital signs were continuously monitored to ensure adequate anesthetic depth and physiological stability, and care was taken to minimize stress and maintain proper ventilation during all interventions.

After hemodynamic assessment, animals previously anesthetized for the procedures were euthanized; blood, lung, and heart tissues were then removed for further analysis.

Blood was collected via the abdominal aorta and transferred into EDTA-containing tubes. Samples were then centrifuged at 900 *g* at 4°C for 10 min, and the supernatant was separated and stored at -80°C for subsequent cytokine measurements.

### Transthoracic echocardiography

Transthoracic echocardiography was performed using Vevo 2100 (VisualSonics, Canada). Mice were anesthetized with 2% isoflurane and 95% oxygen was used to maintain the heart rate at 490±50 bpm. All functional cardiac parameters - tricuspid annular plane systolic excursion (TAPSE), pulmonary artery acceleration time (PAT), pulmonary ejection time (PET), systolic velocity (S'), early diastolic velocity (E'), late diastolic velocity (A'), peak pulmonary function, tricuspid regurgitation - were measured five times, and the means are reported.

### Right heart catheterization (RHC)

The mice underwent terminal right heart catheterization using a right jugular catheter to measure hemodynamics. Briefly, the mice were anesthetized with 2% isoflurane and 95% oxygen inhalation. The pressure-volume catheter (PVR-1035, Millar ADInstruments, USA) was placed into the right ventricle (RV).

### Cytokine analysis

For IL-6, IL-10, and TGF-β1 analysis (R&D System, USA), blood samples were collected in EDTA tubes, centrifuged (900 *g*, 4°C, 10 min), and the supernatant was removed and stored for later determination. Cytokine levels were measured by enzyme-linked immunosorbent assay (ELISA) according to the protocol suggested by the manufacturer. Minimum detection levels for IL-6, IL-10, and TGF-β1 were 15.6 pg/mL.

### Histological examination

Lungs and hearts were collected from PBS-perfused animals and immediately fixed in 10% neutral buffered formalin. Tissues were paraffin-embedded and sectioned, and 3-μm sections were stained with hematoxylin and eosin to evaluate inflammatory infiltrates, granulomas, and/or thickening of vessels/septums, and egg deposition.

Photomicrographs were captured using a light microscope Olympus (Japan) equipped with a digital camera (Software Image Pro-Plus 6.0, Media Cybernetics, USA) at standardized magnifications and consistent exposure settings to ensure comparability across all samples.

### Statistical analysis

Data are reported as means±SD. Comparisons between groups were performed using *t*-test. Person correlation was used to evaluate the association between variables. A P value <0.05 was considered significant. SigmaStat 3.1 (Systat, USA) was used for the analyses.

## Results

**Figure 2:**
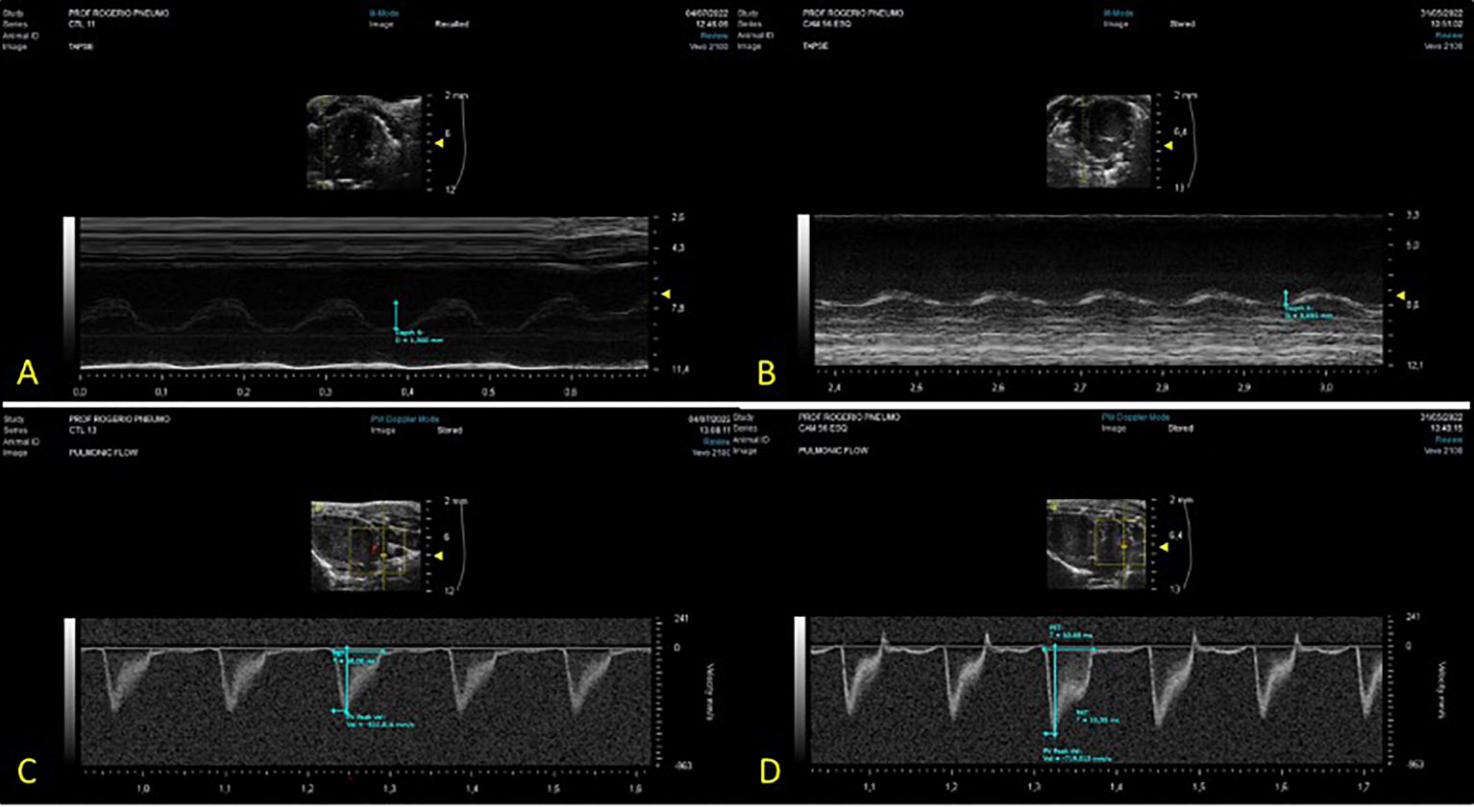
Echocardiography evaluation. **A**, TAPSE (tricuspid annullar plane excursion) of a control animal. **B**, TAPSE of an animal from the *Schistosoma mansoni*-induced pulmonary hypertension (Sch-PH) group showing a reduction. **C**, Pulmonic flow (maximum flow velocity, ejection time, and pulmonary flow acceleration time) of an animal from the control group. **D**, Pulmonic flow of an animal from the Sch-PH group, showing a dagger shape (systolic notch).

**Figure 3:**
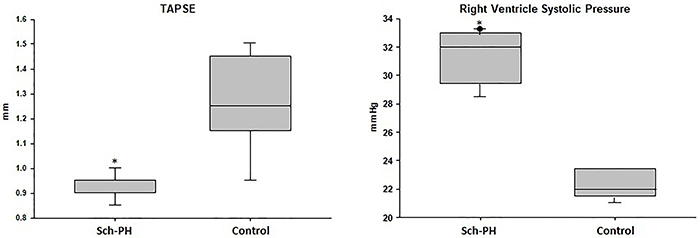
Tricuspid annular plane systolic excursion (TAPSE) and pulmonary artery pressure measurement of mice in the Control group and mice with *Schistosoma mansoni-*induced pulmonary hypertension (Sch-PH group). Data are reported as median and interquartile range. *P<0.05; Mann-Whitney U test.

In the evaluation of echocardiographic parameters, the Sch-PH group animals presented significantly lower TAPSE (0.85±0.15 mm *vs* 1.24±0.18 mm, P<0.001) and PAT/PET ratio (0.20±0.02 *vs* 0.25±0.02, P=0.009) than the control group. For peak pulmonary function (620.9±68.9 mm/s *vs* 512.5±69.7 mm/s), pulmonary regurgitation (389.8±74.9 mm/s *vs* 276.6±70.1 mm/s), and tricuspid regurgitation (718.9±176.6 mm/s *vs* 539.3±132.4 mm/s), animals of the Sch-PH group presented significantly higher values than the control group (P<0.05) (Figures 2 and 3).

In the RVSP analysis, a significant increase (P<0.001) was observed in the Sch-PH group (31.38±1.08 mmHg) compared to controls (22.32±1.08 mmHg) (Figure 3).

**Figure 4:**
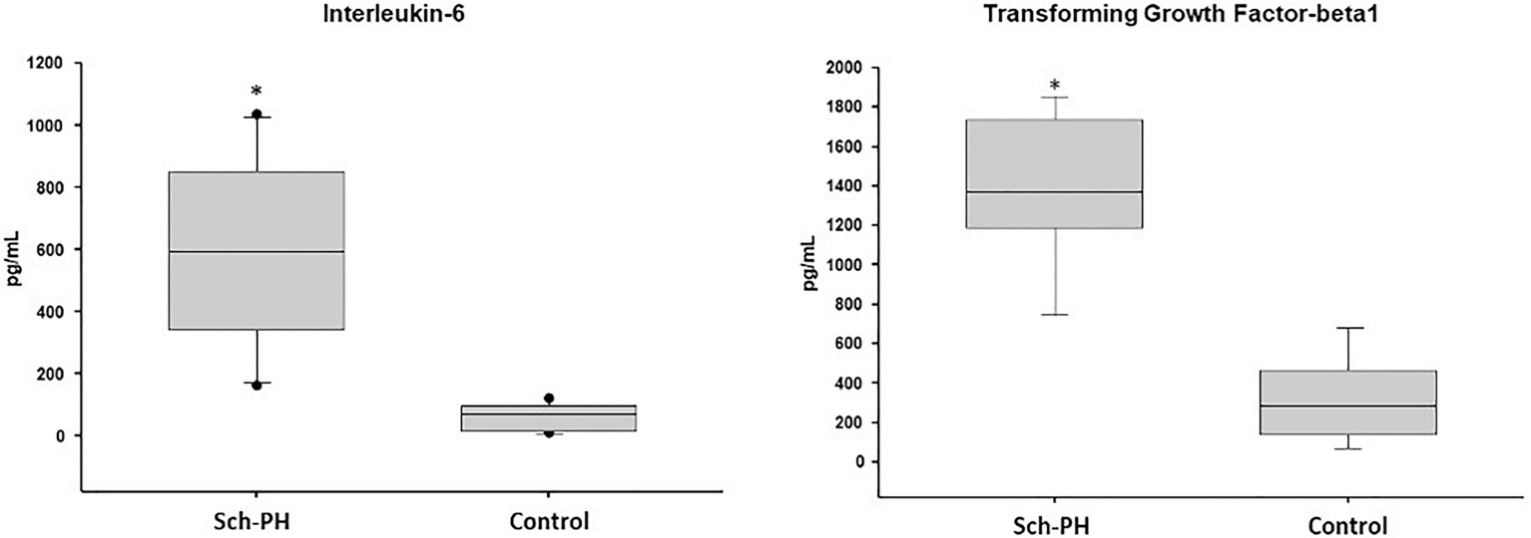
Mice with *Schistosoma mansoni*-induced pulmonary hypertension (Sch-PH group) presented higher interleukin-6 and transforming growth factor-beta1 levels than the Control group measured by ELISA. Data are reported as median and interquartile range. *P<0.05; Mann-Whitney U test.

Serum IL-6 and TGF-β1 levels were significantly higher in animals in the Sch-PH group compared to the control group (577.8±330.5 pg/mL *vs* 59.4±50.1 pg/mL and 1279.1±481.6 pg/mL *vs* 228.8±141.4 pg/mL; P<0.05, respectively) (Figure 4). IL-10 levels were undetectable (<15.6 pg/mL) in the serum of animals of both groups studied. We observed significant correlations between IL-6 and TAPSE (R=-0.730; P=0.010), IL-6 and RVSP measurement (R=0.730; P=0.010), TGF-β1 and TAPSE (R=-0.677; P=0.031), TGF-β1 and RVSP measurement (R=0.927; P<0.001), and IL-6 and TGF-β1 (R=0.644; P=0.044).

**Figure 5:**
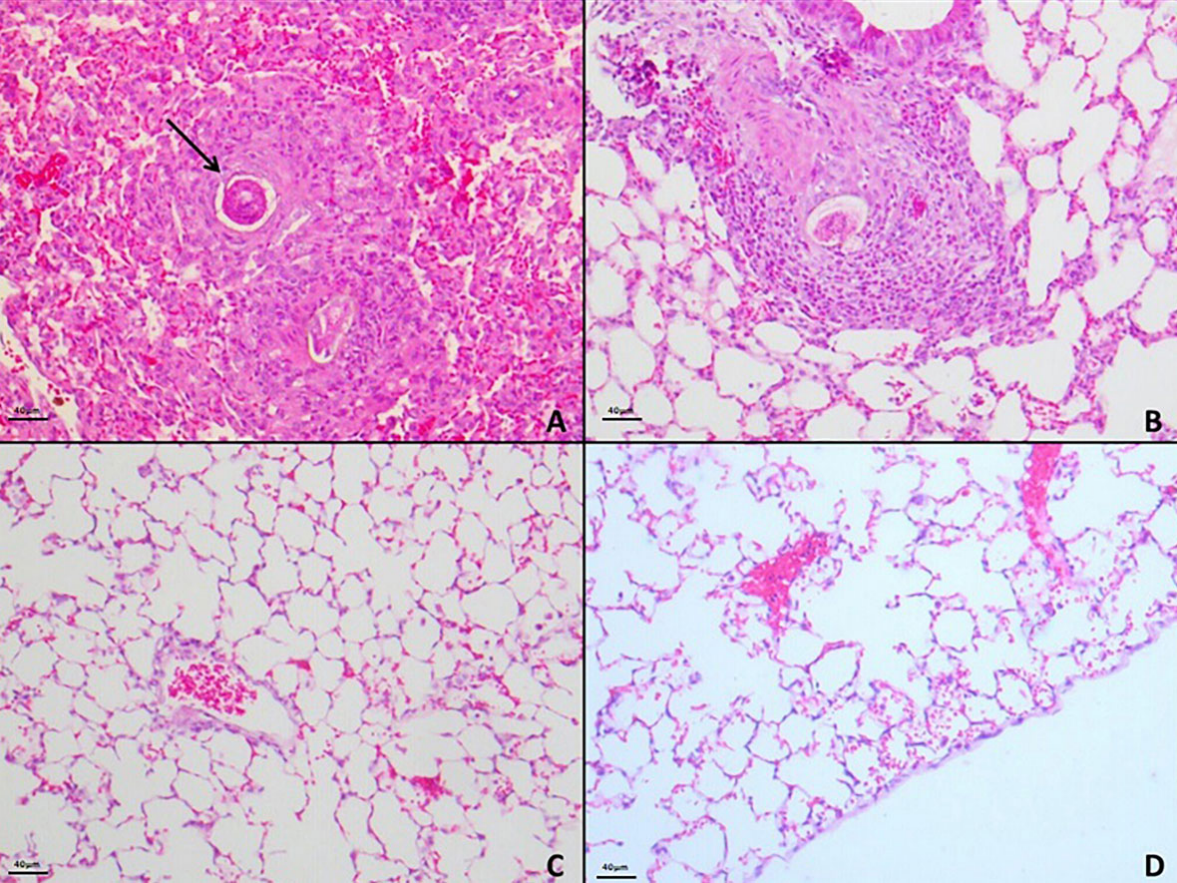
Photomicrographs of lung tissues from animals in the *S. mansoni*-induced pulmonary hypertension (Sch-PH) group showing the presence of *S. mansoni* eggs with inflammatory infiltrate and granulomatous reaction (arrow) (**A**-**D**) stained by H&E (scale bar 40 μm).

In the evaluation of lung tissue after the induction of Sch-PH, diffuse alveolar and perivascular mononuclear and eosinophilic inflammatory infiltrates were observed. Also, granulomatous reactions were observed in the alveolar region and around vessels. Granulomas were eosinophil-rich, and some *S. mansoni* eggs were observed (Figure 5). In the arteries surrounded by granulomas, vessels presented intimal thickening, medial hypertrophy, and fibrosis.

**Figure 6:**
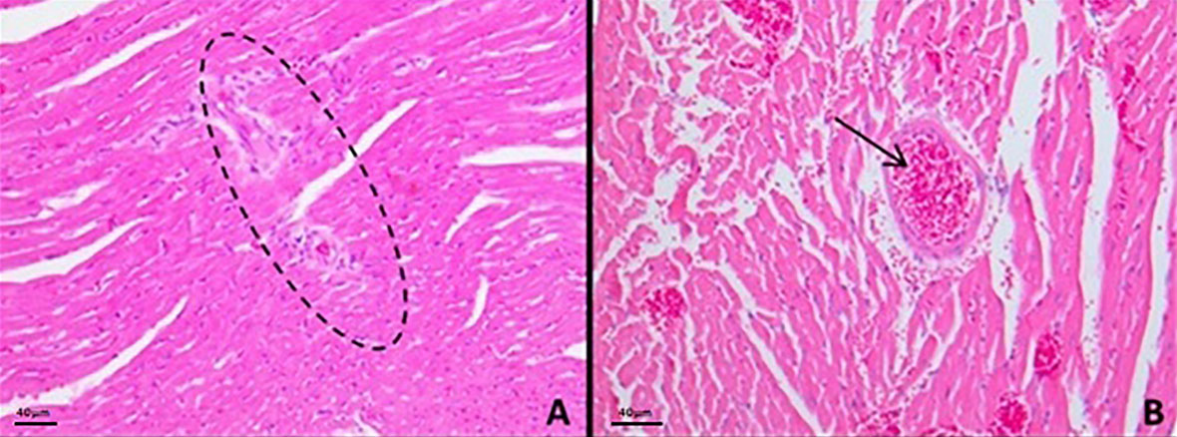
Photomicrographs of cardiac tissue of animals with *Schistosoma mansoni*-induced pulmonary hypertension (Sch-PH) showing (**A**) inflammatory niches throughout the cardiac tissue (ellipse) and (**B**) thickening of the septa (arrow) stained by H&E (scale bar 40 μm).

In the cardiac histological analysis, we observed inflammatory niches throughout the cardiac tissue, proliferation of fibroblast cell nuclei, and thickening of the interstitial connective tissue septa between cardiomyocytes compared to their controls (Figure 6).

## Discussion

Sch-PH is a relevant experimental model to investigate the interplay between parasitic infection, vascular remodeling, and inflammation. In this study, the development of Sch-PH was confirmed by the increased RVSP, echocardiographic evidence of right heart dysfunction, and histopathological alterations in pulmonary arteries adjacent to granulomas containing *S. mansoni* eggs. Furthermore, plasma levels of IL-6 and TGF-β1 were found to be associated with markers of vascular disease, suggesting their involvement in the pathophysiology of Sch-PH, and correlating with disease severity and RV remodeling. To our knowledge, this work is the first report from Brazil to establish this Sch-PH experimental model.

Previous studies have highlighted the role of IL-6 in different forms of PH. In Sch-PH models, IL-6 has been suggested to exert a protective effect in *S. mansoni*-infected animals (7-10,12), whereas in hypoxia- and monocrotaline-induced PH, it plays a central pathogenic role (9,13), reflecting its complex and still uncertain contribution. Steiner et al. (14), in a hypoxia-induced PH model, further showed that IL-6 promotes perivascular remodeling by inducing muscularization of small pulmonary arteries and recruiting inflammatory cells, underscoring its detrimental role in PH. Another relevant pathway is the TGF-β superfamily, currently regarded as a key molecular defect in PAH predisposition and progression (15). TGF-β1 is linked to fibrosis and vascular remodeling. In a rat animal study, the endothelin receptor antagonist macitentan reduced TGF-β1 expression, reinforcing the value of experimental models in identifying novel therapeutic strategies (16). Given these controversial findings, our study showed increased serum IL-6 levels correlated with TGF-β1, as well as with echocardiographic and hemodynamic parameters, underscoring their relevance in Sch-PH pathophysiology. These results suggest that persistent inflammatory and growth factor signaling may drive vascular remodeling and fibrosis. This experimental model thus provides a valuable platform for testing novel IL-targeted therapies and exploring new treatment avenues for Sch-PH.

Elevated circulating IL-6 levels in patients with PH support a mechanistic role for this cytokine in disease pathobiology. Xu et al. (17) reported an inverse correlation between IL-6 and pulmonary function, reinforcing its value as a severity biomarker. In an experimental model using monocrotaline-induced PAH in rats, elevated IL-6 paralleled hemodynamic derangements, and pharmacologic blockade of the IL-6 receptor attenuated PH, underscoring its deleterious effect (18). TGF-β1, another key mediator of the inflammatory response, also correlated with hemodynamic severity in the same model, and crosstalk between IL-6 and TGF-β1 has been implicated in regulating inflammation and vascular remodeling in PH (18-21). Conversely, the anti-inflammatory cytokine IL-10 is downregulated when these markers are upregulated (8,18,22). In our study, serum IL-6 and TGF-β1 correlated negatively with TAPSE and positively with RVSP. Together, these associations link heightened inflammatory and growth factor signaling with worse RV function and higher pulmonary pressures. IL-10 concentrations were low in our samples, mirroring other animal models and supporting IL-10 downregulation in our murine model.

Lapa et al. (23) reported that the heart is among the main organs affected during schistosomal infection, particularly when PAH develops. In our cohort, the infected group exhibited significant right-ventricular dysfunction, corroborating this observation. Owing to the limited feasibility of human assessment, animal models remain central to elucidating pathophysiology and evaluating candidate pharmacotherapies. The Sch-PH model described and adapted by Graham et al. (7,21) provides a pathophysiologically relevant platform to interrogate mechanisms of this severe vascular disease (24). By comparison, traditional hypoxia and monocrotaline models have been criticized for their strong pro-inflammatory bias and incomplete recapitulation of human PAH, whereas the sugen/hypoxia (SuHx) model more closely mimics human vascular remodeling and is now widely used (25,26). Schistosomiasis, however, remains a neglected tropical disease largely endemic to low- and middle-income countries and has limited global research investment (27). Establishing and refining the schistosoma model in Brazil, therefore, offers a unique opportunity to dissect disease mechanisms and, crucially, to test candidate therapies tailored to Sch-PH, advancing translational pathways that are unlikely to emerge from non-parasitic PAH models.

Histologically, it is common to find endomyocardial fibrosis cases in the presence of schistosomiasis infection in humans (28). Cardiac damage due to inflammation may be associated with the fact that toxic products produced by schistosomal granulomas in the liver and intestine are transported to this organ through collateral circulation (27). These findings are directly attributable to *S. mansoni* infection. Nevertheless, in other experimental model settings, such as *S. haematobium* infection, progression to PAH is less pronounced, closely mirroring what is observed in humans, thereby underscoring the specificity and robustness of the *S. mansoni* model for studying Sch-PH (29). *S. mansoni* infection alters the lung structure with an increase in the number of granulomatous reactions, characterized by the grouping of mono- and polymorphonuclear cells in mouse models. The infected animals presented alveolar and/or perivascular infiltrates containing parasitic products, such as periovular granulomas (6). In our study, we observed inflammatory niches throughout the cardiac tissue, proliferation of fibroblast cell nuclei, and thickening of interstitial connective tissue septa between cardiomyocytes when compared to their controls, previously undescribed cardiovascular findings directly attributable to infection of the *S. mansoni* experimental model.

Some points need to be addressed. During *S. mansoni* egg extraction, rigorous precautions were taken to prevent hatching: samples were kept on ice, and heat exposure was avoided. Tail-vein infusion was performed without procedure-related complications. The model using subcutaneous sensitization followed by intravenous egg infusion efficiently induced pulmonary hypertension within a shorter time frame than the cercarial infection model (30), which limits experimental throughput. Given the extended timeline of the cercarial infection approach and its validation in prior international studies, we chose to use the subcutaneous/intravenous egg-infusion model.

This characteristic introduced another limitation in the present study: the eggs were isolated from liver tissue, a standard and readily accessible source for obtaining *S. mansoni* eggs for experimental purposes. We acknowledge that this approach may introduce some bias, as liver-derived eggs can be at different developmental stages or already involved in inflammatory processes, and do not all necessarily contain mature miracidia. However, previous studies employing this experimental model have used the same methodology for egg collection (31). The heterogeneity in egg maturation may be a potential source of variability in the experimental outcomes.

In conclusion, we established a Sch-PH model that showed correlation of IL-6 and TGF-β1 with RV function measured both by echocardiography and invasive measures, a fact not yet systematically explored. This opens the perspective for identification of new therapeutical targets or immunological checkpoints for pulmonary hypertension associated with schistosomiasis.

## Data Availability

The datasets used and/or analyzed during the current study are available from the corresponding author upon reasonable request.
